# Assessment of the Presence of Total Aflatoxins and Aflatoxin B_1_ in Fish Farmed in Two Cameroonian Localities

**DOI:** 10.1155/2020/2506812

**Published:** 2020-09-03

**Authors:** Julie J. Tsafack Takadong, Hippolyte T. Mouafo, Linda Manet, Annick M. B. Baomog, Jorelle J. B. Adjele, Evrard K. Medjo, Gabriel N. Medoua

**Affiliations:** ^1^Centre for Food and Nutrition Research, Institute of Medical Research and Medicinal Plants Studies, PoBox 6163, Yaoundé, Cameroon; ^2^Department of Food Science and Nutrition, National School of Agro-Industrial Sciences, University of Ngaoundéré, PoBox 455, Ngaoundéré, Cameroon

## Abstract

This work aimed at assessing the presence of total aflatoxins (AFs) and aflatoxin B_1_ (AFB_1_) in fish farmed in two Cameroonian localities and the possible origin of that contamination through analysis of fish feeds as well as water and mud collected from the fish farming ponds. Four fish species (kanga, tilapia, catfish, and carp) were collected from two fish farming sites (Mfou and Batié). Mud and water from the farming ponds of the different species and the fish feeds used in these sites were also collected. The samples (34) were analyzed for their levels of AFs and AFB_1_ using the competitive ELISA method. The results obtained showed that all fish tissue contained AFs and AFB_1_. A level of AFs higher than the threshold value recommended by the FDA (20 ppb) was observed in catfish (31.38 ± 0.29 ppb). AFs and AFB_1_ were presented in fish feeds as well as in muds collected from the farming ponds. Catfish was the fish species which mostly bioaccumulated aflatoxins in their tissue. This study presents the state of art on the mycotoxin contamination of fish farmed in some Cameroonian localities and suggests that attention should be paid to the quality of ingredients used to feed fish.

## 1. Introduction

As the world population is increased, the demand for animal proteins has risen, especially those derived from fish because of their high quality in terms of essential amino acid composition; their richness in omega 3 fatty acids, in vitamins (A, D, and B), and in minerals (calcium, iodine, zinc, iron, and selenium); and their low contents in cholesterol and saturated fats [1]. Cameroon is not in rest as fish represents 40% of the protein intake of animal origin by the whole population [2]. To satisfy consumers' demand, government strategies were based on fish importation. However, imported fish does not always satisfy consumers' demand and willingness. The fish is generally imported in frozen form, and the nonrespect of the cold chain during transportation, distribution, storage, and selling is detrimental to its quality. Besides, the demand for fresh fish at an affordable price by some consumers is increasing. To overcome these problems, an alternative approach based on fish farming was introduced [3]. In that order of ideas, the local fish farming sector was boosted by the country who has subscribed to several bilateral projects and multifaceted interventions in order to make fish-farming practices accepted and adopted. Hence, approximately 400,000 tons of fish farmed locally is expected per year in Cameroon [4]. To reach this objective, increase their incomes, and thus satisfy the increasing demand of consumers, most of the fish farmers did not respect the good fish farming practices. They abusively used veterinary drugs, fertilizers, pesticides, and liming materials [5]. In some cases, local food used for fish feeding which is mainly composed of cotton seeds, groundnut flour, maize, fish flour, and animal manure [5] are not properly stored and could result in mycotoxin contamination through mould proliferation [6, 7]. Mycotoxins are toxic secondary metabolites derived from moulds such as the genera *Aspergillus*, *Fusarium*, and *Penicillium* [8]. With low molecular weight, mycotoxins possessed diverse chemical structures [8, 9]. When ingested by living organisms, they can lead to harmful effects [10, 11]. Mycotoxins were reported in the literature as toxic to the kidney, lungs, liver, and the immune, endocrine, and nervous systems [12, 13]. Among mycotoxins, some of them like AFs and AFB_1_ were classified as carcinogenic and mutagenic by the International Agency for Research on Cancer [14].

The presence of AFB_1_ in feed intended to animal consumption was noticed in Southeast Asia by Encarnação and Rodrigues [15]. The authors reported that 55% of animal feed contained AFB_1_ at a mean level of 0.118 mg/kg. In Eastern African countries (Kenya, Tanzania, Rwanda, and Uganda), Marijani et al. [8] reported the presence of mycotoxins in fish feeds and ingredients collected from smallholder farmers. They highlighted that samples from Kenya were the most contaminated with aflatoxins (806.9 *μ*g/kg). In sub-Saharan Africa, only few studies noticed the presence of mycotoxins in fish feeds [16, 17]. Once these mycotoxins are present in fish feed, they can bioaccumulate into the fish tissue and thus represent a risk for human beings [10, 18]. A study previously conducted in our lab highlighted the presence of antibiotic residues in fish farmed in some Cameroonian localities. However, to the best of knowledge, the presence of mycotoxins in locally farmed fish has not yet been studied in Cameroon. Giving that fish are fed through the dispersion of feed in water ponds, we hypothesized that the presence of these mycotoxins in fish tissue could result from a previous contamination of water ponds or from an accumulation of mycotoxins in mud ponds. It appears therefore interesting to assess the presence of mycotoxins in water and mud ponds where fish are farmed. The present study has as objective to assess the presence of AFs and AFB_1_ in fish farmed in two Cameroonian localities and in fish feed used by fish farmers as well as in water and mud ponds.

## 2. Materials and Methods

### 2.1. Study Areas

The present study was conducted in two regions of Cameroon from February 2019 to May 2019. In each region, one site was selected. The first site “Mfou” (3° 96′ 57^″^ N and 11° 93′ 05^″^ E) was located in the district of Mfou, Department of Mefou et Afamba, Centre Region of Cameroon, while the second site “Batié” (5° 18′ 53^″^ N and 10° 19′ 31^″^ E) was located in the district of Batié, Department of Highland, Western Region of Cameroon. The Centre Region of Cameroon was selected because of its climate (temperature between 21°C and 31°C during the hot season and between 18°C and 28°C during the cool season) which favors fish farming. Besides the Centre Region, the Western Region was also chosen for this pilot study because of its cold climate (temperature between 16°C and 20°C) which is favorable to the farming of some specific species of fish.

### 2.2. Fish Farming Ponds

The two sites retained for this study contain more than four farming ponds. The mean area of the different farming pond was approximately 600 m^2^. In the fish farming site “Mfou,” the species kanga and tilapia are farmed in the same pond while the species catfish is farmed alone in another pond of the same farming site. That association of fish species was for controlling the reproduction and increasing the final yield [19]. In the fish farming site “Batié,” only carp is farmed in different ponds.

### 2.3. Sampling

Three species of fish were collected in the fish farming site “Mfou.” They were tilapia (*Oreochromis niloticus*), African catfish (*Clarias gariepinus*), and kanga (*Heterotis niloticus*). These species represent the main fish farmed in the Centre Region of Cameroon. Another species of fish for which the optimal growth conditions required low temperature, namely, common carp (*Cyprinus carpio*) was collected in the farming site “Batié.” Regarding the sampling procedure, the method of the European Commission [20] was used. Briefly, for each species, fish were withdrawn from the farming pond and six fish of about 500 g each (total of 3 kg) aged between 8 and 12 months were collected, iced immediately, and transported in an icebox containing frozen blocks to the laboratory. Given that the aim of this study was to assess the level of mycotoxins in fish tissue and identify the possible sources of contamination of these fish by mycotoxins, besides fish, samples of fish feed (1 kg each), water pond (1 L each), and mud ponds (1 kg each) were also collected following the method of the European Commission [20]. The samples were labelled and transported together with fish samples to the laboratory where the analyses were performed.

### 2.4. Fish Samples Processing

Upon arrival at the lab, fish were descaled, neatly eviscerated, and washed with tap water in a cold environment. Afterwards, fish were filleted and the fillets were mixed and crushed using a mincer (Black & Decker®, England). The pastes obtained were homogenized, divided into several aliquots of 10 g each, and kept at -20°C until the day of experimentation.

### 2.5. Determination of Total Aflatoxin (AFs) and Aflatoxin B_1_ (AFB_1_) in the Samples

The levels of AFs and AFB_1_ in the different samples were assessed using the quantitative method ELISA (enzyme-linked immune sorbent assay). The MaxSignal® ELISA Test kits for AFs and AFB_1_ used in this study were provided by BIOO Scientific Corp (USA).

#### 2.5.1. Extraction of Mycotoxins

The extraction process was carried out following the protocol defined by the manufacturer for each type a matrix. Regarding fish paste, 2 g of sample was mixed with 8 mL of 87.5% methanol (HPLC grade, Sigma, Germany) and vortexed for 10 min (Vortex Genius 3, IKA, Germany). The mixture was then centrifuged at 4000 g for 10 min (Centrifuge Rotofix 32 A, Germany), and the supernatant was kept for analysis. For the fish feed sample, 5 g was mixed with 25 mL of 70% methanol. The obtained solution was vortexed for 10 min and centrifuged (4000 g, 10 min), and the supernatant was collected.

Concerning water and mud collected from farming ponds, 1 g was introduced into a tube containing 1 mL of extraction solution (methanol/extraction buffer 1X: 6 : 4, v/v). The mixture was vortexed for 1 min and centrifuged (4000 g, 5 min), and the supernatant was collected for assay.

#### 2.5.2. Competitive ELISA and Plates Reading

The collected supernatants were used for the Competitive Direct ELISA following the manufacturer's instruction. Plates were immediately read at 450 nm using an automated microplate reader (EL ×800, BIOTEK, Instruments Inc., Winooski, VT, USA). AFs and AFB_1_ standards at 0, 0.05, 0.25, 0.75, 2.5, and 10 ppm were used to plot the calibration curves (*r*^2^ above 0.98). The calibration curves were used to calculate the contents of AFs and AFB_1_ in the different samples. Samples with AFs and AFB_1_ levels below the limit of detection as specified by the kits' manufacturer (0.01 ppb for water and mud and 1 ppb for fish and feed samples) were considered as containing no detectable AFs or AFB_1_.

### 2.6. Statistical Analyses

Experiments were performed in triplicates. Data obtained were expressed as means ± standard deviation and submitted to analysis of variance (ANOVA). The Duncan multiple range test was used to compare the means at *p* < 0.05. These analyses were performed with the software Statgraphics Centurion XV version 16.1.18 (StatPoint Technologies, Inc., USA).

## 3. Results

### 3.1. Total Aflatoxins and Aflatoxin B1 Levels in Fish Samples

Table 1 presents the minimum and maximum contents of AFs and AFB_1_ found in fish tissue collected from the farming site “Mfou” and “Batié.” Both AFs and AFB_1_ were detected in the samples analyzed at a level which significantly (*p* < 0.05) varies with the fish species. Regarding fish species, the levels of AFs and AFB_1_ vary significantly (*p* < 0.05) from a species to another. Catfish was the most contaminated fish with an AFB_1_ content ranging from 1.81 to 15.69 ppb and an AF content which varies from 3.62 to 31.38 ppb. The lowest levels of AFs (0.21 ppb) and AFB_1_ (0.10 ppb) were observed with the species kanga. For a fish species, a significant variation (*p* < 0.05) of the AF and AFB_1_ contents were noticed. The highest recorded value was 40 times higher than the lowest one in the case of tilapia, 50 times in the case of kanga, 8.6 times in the case of catfish, and 2.16 times in the case of carp.

### 3.2. Total Aflatoxin and Aflatoxin B1 Levels in Fish Feed, Mud, and Water Samples

In order to identify the possible source of contamination of fish with AFs and AFB_1_, the presence of these mycotoxins in farming pond environments such as water and mud as well as their presence in food used to feed fish were assessed.

Regarding the mud samples, Table 2 shows that AFs and AFB_1_ were presented in all samples analyzed at levels which significantly (*p* < 0.05) vary with the species of fish farmed in the pond. The mud where carp were farmed contains more AFs (31.55 ppb) and AFB_1_ (15.77 ppb) than those where kanga, tilapia, and catfish were farmed. The least contaminated mud was the one that originated from the farming pond of catfish (1.03 ppb for AFB_1_ and 2.06 ppb for AFs). When the fish farming site is considered, it clearly appears from Table 2 that the mud coming from the farming site “Batié” was more contaminated than those coming from the farming site “Mfou.” However, there was no direct relationship between the presence of AFs and AFB_1_ in mud and their presence in fish tissue. The level of AFs and AFB_1_ in carp was very low compared to their level in mud. An opposite phenomenon was observed with the other fish species where mud coming from the farming ponds were least contaminated than fish tissue.

Concerning water pond samples, AFs and AFB_1_ were present in all samples at concentrations below the detection limit which is 0.1 ppb as specified by the kit manufacturer. The most contaminated water was those collected from the pond of catfish (0.09 ppb). This result was positively associated with the level of these mycotoxins in fish tissue as catfish scored the highest level of contamination in both AFs and AFB_1_. However, the mud collected from the same catfish pond scored the lowest level of AFs and AFB_1_. In the farming pond of kanga and tilapia, the results obtained in this study show that fish tissue was more contaminated than water and mud.

Feeds consumed by fish were also assessed for their AF and AFB_1_ contents, and the results are presented in Table 2. Feeds contain 1.82 ppb of AFB_1_ and 3.64 ppb of AFs. Generally, it clearly appears that the levels of AFB_1_ and AFs in feed were lower than their levels of fish.

## 4. Discussion

AFs and AFB_1_ are toxins produced mainly by *Aspergillus flavus*, *A. parasiticus*, *A. nomius*, *A. arachidicola*, and in some cases by *Emericella astellata*, *E. venezuelensis*, and *E. olivicola* [21]. Their presence in food even at low concentrations is harmful to human beings as they can be bioaccumulated into tissue [10, 18, 22]. In this study, fish farmed in Cameroonian localities and locally consumed by the population were assessed for the occurrence of AFs and AFB_1_ (which is the most toxic aflatoxin chemotype) [8] in their tissue. The results showed that these mycotoxins were present in all fish samples at concentrations which significantly vary from one fish species to another. Yiannikouris and Jouany [23] also notified in their studies that the occurrence of aflatoxins in fish samples depends on the sensibility of the fish. Tilapia was the less contaminated fish species while catfish was the most contaminated one (Table 1). According to cited reports [24], catfish (40.40 ppb) was also more contaminated than tilapia (15.11 ppb). This difference in the contamination could be explained by the variability of metabolic activities from one species to another. Furthermore, the amount of contaminated feed daily ingested by the fish could also justify the difference observed. Similar observations were noticed by Binder et al. [25] in their studies on the aflatoxin contamination of fish samples. The significant difference observed between the AF and AFB_1_ concentrations in kanga and tilapia which are both farmed in the same pond also confirmed the hypothesis that the metabolic activities involved in the storage and/or elimination of mycotoxins in fish tissue vary with the fish species. The presence of AFs and AFB_1_ in fish farmed in Cameroonian localities is of greatest concern because they can lead to negative effects on the development of fish such as stunting, liver damage, immunosuppression [26], decrease in growth performance, and increase susceptibility to disease and high mortality [27]. Rahman et al. [28] pointed out the significant reductions in the total serum proteins, albumins, and globulins caused by the presence of AFB_1_ in Nile tilapia. Murjani [10] also highlighted in their studies the carcinogenic effect of AFB_1_ on fish such as salmon, rainbow trout, channel carp, tilapia, and guppy. All these side effects on fish might have as consequences such as an increase of the economic losses (due to a decrease in productivity and higher mortality rates) [29, 30] and an increase in the risk of incidence of adverse effects of mycotoxins on human beings.

Considering the recommendation of the Food and Drug Administration [31] in relation to the maximum level of AFs and AFB_1_ in food intended for human consumption (20 ppb), some samples of catfish were not suitable for human feeding. This result suggests that attention should be paid to the bioaccumulation potential of aflatoxins by that species of fish.

The most contaminated mud pond was the one where carp was farmed. This result could be explained by the feeding frequency of fish as observed in the site “Batié.” In fact, in the site “Batié,” fish were fed every day compared to the site “Mfou” where they were fed once per week. In these conditions, it clearly appears that the feed unconsumed daily, if contaminated with AFs and AFB_1_, can precipitate to the bottom of the pond and accumulate in the mud. However, the levels of AFs and AFB_1_ in mud samples were not associated with their levels in carp tissue. This observation shows that the main source of AFs and AFB_1_ in fish tissue is the feed that they ingested.

AFs and AFB_1_ were found in fish feed samples collected from the fish farming sites (1.82 ppb for AFB1 and 3.64 ppb for AFs). Their levels in fish feed were lower than the threshold value recommended by the European Commission for undesirable substances in animal feed which is 20 ppb [32]. Compared to the literature, the level of AFB_1_ in fish feeds observed in this study was low. Marijani et al. [8] noticed a level of AFB_1_ ranging from 2 to 806 ppb in fish feeds and ingredients collected from smallholder farmers in Kenya, Tanzania, Rwanda, and Uganda. In Iran, Santacroce et al. [27] found an AFB_1_ level in fish feeds ranging from 0.46 to 68.5 ppb. In Brazil, Barbosa et al. [30] reported that the AFB_1_ level of fish feed ranged from 1.83 to 67.35 ppb. The presence of these toxins in fish feed samples as observed in the present study could be explained either by the use of ingredients previously contaminated with AFs or AFB_1_ to prepare these feeds or through poor storage conditions which had favored the growth of fungi and thus the production of mycotoxins [8]. Mahfouz and Sherif [33] also noted that storage practices and processing methods of feed are favorable to fungal growth and production of mycotoxins. Furthermore, the data gathered from this study showed that the levels of AFB_1_ and AFs in feeds are lower than their level in fish tissue. This phenomenon could be justified by the bioaccumulation principle of mycotoxins in fish tissue as time passed. However, the mean concentration of AFs obtained in this study was lower than that reported by Mwihia et al. [34] in fish feed used in fish farming activities in Kenyan localities (1.8 to 39.7 ppb). This difference could result from the level of contamination of the different ingredients used in feed preparation as well as the storage conditions of feeds [8].

Independent of the sampling site, the different water ponds analyzed showed a level of contamination of less than 0.1 ppb. This result can be justified by the weak solubility of AFs and AFB_1_ in water. According to the literature, aflatoxins are slightly soluble in water [35]. Hence, aflatoxins which have more affinity with organic compounds will coaggregated with the rest of the insoluble matter and precipitated to the bottom of the pond. Furthermore, the continuous replacement of water during the pond cleaning or fishing processes could also explain the weak level of AFs and AFB_1_ obtained in the present study.

## 5. Conclusions

The present study showed that fish farmed in two Cameroonian localities contained AFs and AFB_1_. The most contaminated species is catfish, and the less contaminated one is tilapia. Only catfish presented a level of AFs and AFB_1_ higher than the recommended norm for these toxins (20 ppb). For fish farmed in the same pond, the level of contamination with AFs and AFB_1_ varies with the fish species. The food used to feed fish contained AFs and AFB_1_ and appears as the main source from which fish are getting contaminated. AFs and AFB_1_ were present in the mud of ponds where fish are farmed at the concentration which depends on the fish feeding frequency. This study presents the state of art on the mycotoxin contamination of fish farmed in two Cameroonian localities and suggests that attention should be paid to the quality of ingredients used for fish feeding especially for the case of catfish which seems to accumulate more mycotoxins in its tissue.

## Figures and Tables

**Table 1:** Total aflatoxins (AFs) and aflatoxin B_1_ (AFB_1_) contents (ppb) of fish samples collected from the different farming ponds.

Mycotoxins		Carp	Tilapia	Kanga	Catfish
AFs	Minimum	6.56 ± 0.30^c^	0.26 ± 0.07^a^	0.21 ± 0.01^a^	3.62 ± 0.01^b^
	Maximum	17.35 ± 2.91^c^	8.64 ± 0.03^a^	11.46 ± 0.39^b^	31.38 ± 0.29^d^
	Means	11.93 ± 0.02^c^	4.48 ± 0.04^a^	5.84 ± 0.01^b^	17.72 ± 0.31^d^
	Norm	20			
AFB_1_	Minimum	3.17 ± 0.01^c^	0.13 ± 0.03^a^	0.10 ± 0.00^a^	1.81 ± 0.00^b^
	Maximum	8.67 ± 1.45^b^	4.32 ± 0.01^a^	5.59 ± 0.00^a^	15.69 ± 0.14^c^
	Means	5.87 ± 0.07^c^	2.23 ± 0.01^a^	2.82 ± 0.03^b^	8.81 ± 0.08^d^
	Norm	20			

*n* = 34. Values are expressed as mean ± standard deviation. Values bearing the same superscript letter on the same row are not significantly different at *p* < 0.05 according to the Duncan multiple range test. Norm = mycotoxin regulatory guidance of the Food and Drug Administration [21].

**Table 2:** Levels (ppb) of total aflatoxins and aflatoxin B_1_ in water, mud, and fish feed samples collected from the different farming ponds.

Samples	Specification	AFB_1_ (ppb)	AFs (ppb)
Mud	Mud from kanga and tilapia pond	2.10 ± 0.04^b^	4.21 ± 0.85^b^
	Mud from carp pond	15.77 ± 0.00^c^	31.55 ± 0.01^c^
	Mud from catfish pond	1.03 ± 0.39^a^	2.06 ± 0.79^a^
Water	Water from kanga and tilapia pond	0.03 ± 0.00^a^	0.07 ± 0.01^a^
	Water from carp pond	0.04 ± 0.02^a^	0.08 ± 0.00^a^
	Water from catfish pond	0.07 ± 0.00^b^	0.09 ± 0.01^a^
Fish feed	Samples	1.82 ± 0.35	3.64 ± 0.72
	Norm	20	20

*n* = 34. Values are expressed as mean ± standard deviation. Values bearing the same superscript letter on the same column are not significantly different at *p* < 0.05 according to the Duncan multiple range test. Norm = European Commission [32].

## Data Availability

Upon request, the data used in this study are available from the corresponding author.

## References

[1] Mohanty B. P., Mahanty A., Ganguly S., Mitra T., Karunakaran D., Anandan R. (2019). Nutritional composition of food fishes and their importance in providing food and nutritional security. *Food Chemistry*.

[2] BUCREP (2012). *Rapport national sur l’état de la population édition 2012. Enjeux et défis d’une population de 20 millions d’habitants au Cameroun en 2012*.

[3] Pouomogne V., Pemsl D. E. (2008). *Recommendation domains for pond aquaculture. Country case study: development and status of freshwater aquaculture in Cameroon, World Fish Center Studies and Reviews No. 1871. Penang*.

[4] MINEPIA (Ministry of livestock, fisheries and animal Industries) & FAO (Food and Agricultural Organization) (2009). *Sectorial review of aquaculture Cameroon*.

[5] Ntsama I. S. B., Tambe B. A., Takadong J. J. T., Nama G. M., Kansci G. (2018). Characteristics of fish farming practices and agrochemicals usage therein in four regions of Cameroon. *Egyptian Journal of Aquatic Research*.

[6] El-Sayed Y. S., Khalil R. H. (2009). Toxicity, biochemical effects and residue of aflatoxin B_1_ in marine water-reared sea bass (*Dicentrarchus labrax* L.). *Food and Chemical Toxicology*.

[7] Embaby E. M., Ayaat N. M., Abd El-Galil M. M., Abdel-Hameid N. A., Gouda M. M. (2015). Mycoflora and mycotoxin contaminated chicken and fish feeds. *Middle East Journal of Applied Science*.

[8] Marijani E., Wainaina J. M., Charo-Karisa H. (2017). Mycoflora and mycotoxins in finished fish feed and feed ingredients from smallholder farms in East Africa. *Egyptian Journal of Aquatic Research*.

[9] Nguegwouo E., Tchuenchieu A., Mouafo H. T., Fokou E., Medoua N. G., Etoa F. X. (2018). An overview of some major mycotoxins in food and their detection methods. *Nutrition and Food Toxicology*.

[10] Murjani G. (2003). *Chronic aflatoxicosis in fish and its relevance to human health*.

[11] Alshannaq A., Yu J. H. (2017). Occurrence, toxicity, and analysis of major mycotoxins in food. *International Journal of Environmental Research and Public Health*.

[12] CAST (Council for Agricultural Science and Technology) (2003). Mycotoxins: risks in plant, animal, and human systems. *Task Force Report*.

[13] Caruso D., Talamond P., Moreau Y. (2013). Mycotoxines et pisciculture: un risque oublié?. *Cahier Agricole*.

[14] IARC (International Agency for Research on Cancer) (1993). Evaluation of carcinogenic risks of chemical to humans. *Some naturally-occurring substances: Food Items and Constituents. Heterocyclic Aromatic Amines and Mycotoxins*.

[15] Encarnação P., Rodrigues L. (2011). The threat of mycotoxins in aqua feeds. *Aquaculture Asia Pacific Magazine*.

[16] Bryden W. L. (2012). Mycotoxin contamination of the feed supply chain: implications for animal productivity and feed security. *Animal Feed Science and Technology*.

[17] Njobeh P. B., Dutton M. F., Åberg A. T., Haggblom P. (2012). Estimation of multi-mycotoxin contamination in South African compound feeds. *Toxins*.

[18] Shephard G. S. (2008). Risk assessment of aflatoxins in food in Africa. *Food Additives & Contaminants: Part A*.

[19] Bogne Sadeu C., Mikolasek O., Pouomogne V., Eyango M. T. (2013). The use of wild catfish (*Clarias* spp.) in combination with Nile tilapia (*Oreochromis niloticus* L.) in Western Cameroon: technical performances, interests, and limitations. *Journal of Applied Aquaculture*.

[20] EC (European Commission) (2006). Laying down the methods of sampling and analysis for the official control of the levels of mycotoxins in foodstuffs, Commission Regulation No 401.2006. *Official Journal*.

[21] Abrunhosa L., Morales H., Soares C. (2013). A review of mycotoxins in food and feed products in Portugal and estimation of probable daily intakes. *Critical Reviews in Food Science and Nutrition*.

[22] Nguegwouo E., Tchuenchieu A., Mouafo H. T. (2019). Mycotoxin contamination of food and associated health risk in Cameroon: a 25-years review (1993-2018). *European Journal of Nutrition & Food Safety*.

[23] Yiannikouris A., Jouany J. P. (2002). Mycotoxins in feeds and their fate in animals: a review. *Animal Research*.

[24] Wang X., Wang Y., Li Y. (2016). Response of yellow catfish (*Pelteobagrus fulvidraco*) to different dietary concentrations of aflatoxin B_1_ and evaluation of an aflatoxin binder in offsetting its negative effects. *Ciencias Marinas*.

[25] Binder E. M., Tan L. M., Chin L. J., Handl J., Richard J. (2007). Worldwide occurrence of mycotoxins in commodities, feeds and feed ingredients. *Animal Feed Science and Technology*.

[26] Fallah A. A., Pirali-Kheirabadi E., Rahnama M., Saei-Dehkordi S. S., Pirali-Kheirabadi K. (2014). Mycoflora, aflatoxigenic strains of A*spergillussection* Flaviand aflatoxins in fish feed. *Quality Assurance and Safety of Crops & Foods*.

[27] Santacroce M. P., Conversano M. C., Casalino E. (2008). Aflatoxins in aquatic species: metabolism, toxicity and perspectives. *Reviews in Fish Biology and Fisheries*.

[28] Abdel Rahman A. N., Abdellatief S. A., Mahboub H. H. H. (2017). Protection of Nile tilapia, Oreochromis niloticus from aflatoxin B1 toxicity by dietary supplementation with Fennel essential oil and *Saccharomyces cerevisiae*. *Egyptian Journal of Aquatic Research*.

[29] Rodrigues I., Handl J., Binder E. M. (2011). Mycotoxin occurrence in commodities, feeds and feed ingredients sourced in the Middle East and Africa. *Food Additives and Contaminants: Part B*.

[30] Barbosa T. S., Pereyra C. M., Soleiro C. (2013). Mycobiota and mycotoxins present in finished fish feeds from farms in the Rio de Janeiro State, Brazil. *International Journal of Aquatic Research*.

[31] Food and Drug Administration (FDA) U. S., FDA (Food and Drug Administration) (2011). U.S. Food and Drug Administration (FDA) mycotoxin regulatory guidance.

[32] EC (European Commission) (2002). European Commission Directive 2002/32/EC of the European parliament and of the council of 7^th^ May 2002 on undesirable substances in animal feed. *Official Journal of European Communities*.

[33] Mahfouz M. E., Sherif A. H. (2015). A multiparameter investigation into adverse effects of aflatoxin on *Oreochromis niloticus* health status. *The Journal of Basic & Applied Zoology*.

[34] Mwihia E. W., Mbuthia P. G., Eriksen G. S. (2017). Occurrence and levels of aflatoxins in fish feeds and their potential effects on fish in Nyeri, Kenya. *Toxins*.

[35] Brera C., De Santis B., Debegnach F., Miraglia M., Picó Y. (2008). Mycotoxins. *Comprehensive Analytical Chemistry*.

